# Supplementary material for: Community composition exceeds area as a predictor of long-term conservation value
Source: PLoS Comput Biol. 2023 Jan 30;19(1):e1010804. doi: 10.1371/journal.pcbi.1010804 (PMC9946215; doi:10.1371/journal.pcbi.1010804)
Supplement: S1 Text — (PDF) [file pcbi.1010804.s001.pdf]

# Supporting information

## A Harvesting experiment and the CAR

Following [1] we computed an $S \times S$ spatially unresolved competition matrix $\mathbf{C}$ that summarised biotic interactions at the regional scale. For this we constructed a spatially unresolved Lotka-Volterra system,

$$\frac{dB_i}{dt} = \left( \rho_i - \sum_{j=1}^{S} C_{ij} B_i \right) \cdot B_i, \tag{S1}$$

where $B_i$ represents total biomass of species $i$, as an approximation of the spatially resolved model. The regional scale interaction coefficients $C_{ij}$ were then estimated using a computational harvesting experiment, which assumes that interaction strengths can be inferred from the changes in regional abundances that result from controlled changes in the regional abundances of harvested species [2].

For the unresolved model, Eq. S1, the harvesting of species $i$ at a rate $h$, represented by substituting $\rho_i - h$ for $\rho_i$ in Eq. S1, produces a shift in the equilibrium biomasses given by

$$\sum_{y} \left( B_{jy} - B^*_{jy} \right) = \Delta B_j = -C^{-1}_{ij} h. \tag{S2}$$

where $\mathbf{B}^*$ represents the unharvested equilibrium. Near an equilibrium $B^*_{ix}$ of the full metacommunity model,

$$\frac{dB_{ix}}{dt} = \left( \sum_{jy} J_{ixjy} (B_{jy} - B^*_{jy}) \right) - hB^*_{ix}, \quad \text{and} \tag{S3a}$$

$$\frac{dB_{kx}}{dt} = \left( \sum_{jy} J_{kxjy} (B_{jy} - B^*_{jy}) \right) \quad \text{for } k \neq i \ . \tag{S3b}$$

where $h$ is the harvesting rate, and $\mathbf{J}$ is the numerically estimated $SN \times SN$ Jacobian of the full metacommunity model. We vectorize the matrix $\mathbf{B}$ (denoted $\vec{\mathbf{B}}$) to match the dimensionality of the spatially resolved Jacobian, and the write the equilibrium condition for Eq. S3a as

$$\mathbf{J}(\vec{\mathbf{B}} - \vec{\mathbf{B}}^*) - \vec{\mathbf{H}} = 0, \tag{S4}$$

where the elements of vector $\vec{\mathbf{H}}$ are $hb^*_{jy}$ for $j = i$ and 0 otherwise. From this, we obtain

$$h^{-1} \left( \vec{\mathbf{B}} - \vec{\mathbf{B}}^* \right) = \mathbf{J}^{-1} h^{-1} \vec{\mathbf{H}}. \tag{S5}$$

The left hand side of Eq. S5 represents the local change in biomasses due to the harvesting of the focal species $i$ per unit $h$. From Eq. S5 we compute the change in total biomass $\Delta B_j = \sum_x B_{jx} - B^*_{jx}$ for each species $j$, which gives row $i$ of $\mathbf{C}^{-1}$ (Eq. S2). Iterating over all species $i = 1 \ldots S$, we computed $\mathbf{C}^{-1}$ and from this the spatially unresolved interaction matrix $\mathbf{C}$. This method requires that the metacommunity is at equilibrium with respect to biomass. In practice, this assumption is violated by the onset of autonomous compositional turnover for large $N$. For this reason we restricted our analysis to a range of $N$ for which autonomous turnover is absent or low.

The approximate functional form of the CAR and the scaling relationships between its exponents $v$ and $v'$, the exponent of the SAR $z$ and the abiotic niche width $w$ can be studied by assembling metacommunities of various numbers of sites (total area) and computing the mean regional-scale interaction coefficients $\overline{C_{ij}}$ using the harvesting

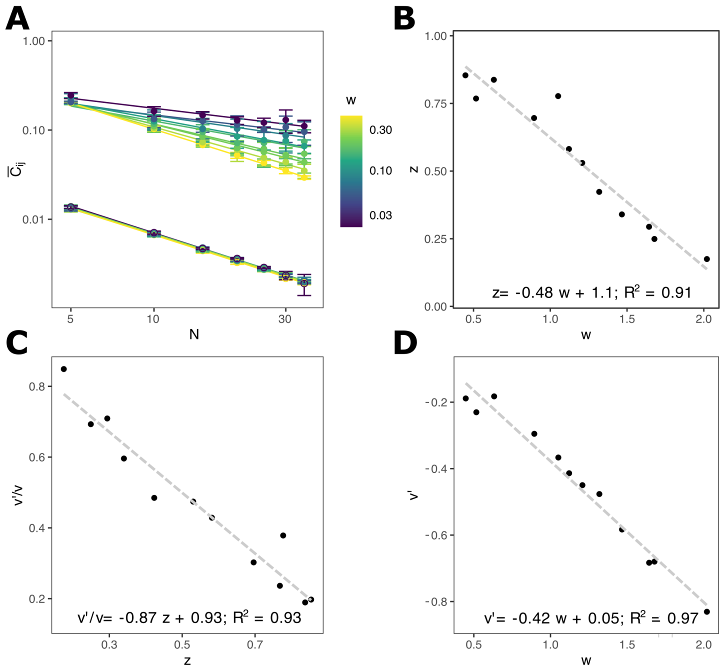


**Fig A. Competition area relations in the LVMCM.** Assembling metacommunities with different total area reveals a power-law decay in average regional scale competitive interaction coefficients (A). We show the CAR for random metacommunities of $5 \leq N \leq 35$ sites for a range of niche widths $w$. The area dependence of mean inter-specific interaction strengths are largely unimpacted by niche width. In contrast there is a substantial effect of $w$ on the exponent $v'$ for intra-specific interactions. Linearly regressing the exponent $z$ of the species-area relation (SAR) against niche width $w$ (B), $v'/v$ against $z$ (C) and $v'$ against $w$ (D) offers some indication of the mechanisms through which effective heterogeneity impacts the SAR and inform the scaling of interactions in our model. In B-D, the exponents $z$, $v$ and $v'$ were computed by assembling metacommunities for given $w$ and $N = 5, 10, 15, 20, 25, 30, 35$ sites in 10-fold replication, such that each point in B-D represents an regression across 70 independent simulations.

approach (Fig Aa). The exponents $v$ and $v'$ depend on model parameters, in particular $w$, which strongly affects species' range sizes (we found the effect of dispersal length to be weak by comparison). Future developments in the theory of LV metacommunities are likely to reveal close relationships between the CAR, the SAR ($S \propto a^z$) and the effective heterogeneity of the landscape (here determined mainly by $w$). In lieu of analytic results, we report the following three statistical relationships between these key quantities in Fig Ab-d. i) The exponent of the SAR $z$ decays with abiotic niche width $w$, suggesting greater abiotic heterogeneity (smaller niche width) results in a steeper SAR. This is consistent with previous results [1] and qualitatively matches the observation of a steep phase in the empirical SAR at the highest spatial scales (greatest effective heterogeneity) [3–5]. ii) The inequality $v'/v \geq 1$ holds over the examined parameter space and further we find the approximate relationship $v'/v \approx 1 - z$. This suggests a pivotal role for the *ratio* between the sensitivity of inter- and intra-specific interaction coefficients to area in determining the exponent of the SAR. Finally, iii) the rate of decay of the intra-specific interaction coefficient with area $v'$, and abiotic niche

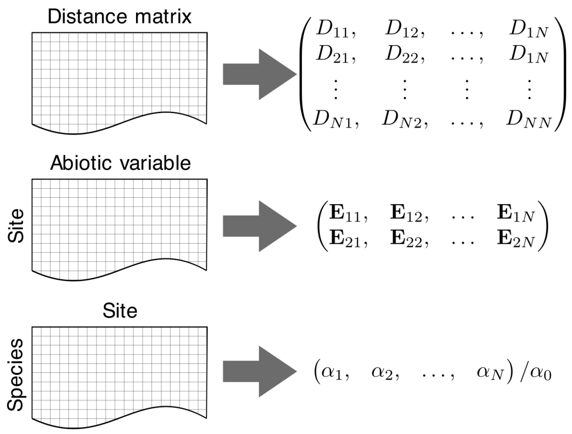


**Fig B. Extracting data for fitting to empirical observation.** To fit the LVMCM to empirical observation we extract spatial coordinates, scaled to a square of longest side length $\sqrt{20}$ so that parameter values correspond to those used in the random metacommunity simulations. From the species by site presence-absence table, we extract the local site richness, presumed to scale with site area (quality). This is then used to generate $\lambda$, and $\lambda'$ which scale the interaction coefficients $\mathbf{A}_{ij}$ so that the simulated metacommunity reproduces the observed distribution in $\alpha$-diversity. From the available environmental metadata we extract the first two principle components and input these as the vectors $\mathbf{E}_k$ in the simulation.

width $w$ are negatively correlated. When abiotic niche widths are very small, intra-specific interaction coefficients are largely insensitive to total area ($v'$ approaches 0), since species ranges are primarily limited by the environment and not by total area. In contrast, when niche widths are large and species distributions can encompass much of the landscape, effective intra-specific interactions decay more rapidly with total area ($v'$ approaches $v$, Fig Aa). The linear associations revealed in Fig Ab-d may hold only for a subset of the parameter space of the model, however this subset includes the parameter combinations that we find to best fit empirical data.

We assume that accumulation of diversity with area within sites is linear, that is, that $z = 1$. This simplification is biologically reasonable given the tendency for the initial phase of empirical SARs to be linear. From the relationships between the key quantities summarised in Fig A we can therefore set the exponents of the CAR in this linear phase as $v = -0.59$, $v' = -0.04$. These were then used to parameterise equations (3), rescaling local site interaction matrices and, by extension, total biomass (area).

## B Detailed procedure for fitting to empirical data

Our approach to fitting the LVMCM to empirical species-by-site tables is as follows: We determined for each data set the Universal Transverse Mercator coordinates of each site, the two principal components of a multivariate distribution of environmental variables scaled to mean zero and unit variance, and the species richness recorded at each site (Fig B). The first two are input directly into the model. For better comparison to the parameters used above, we scaled the spatial network such that it occupies an arena of which the longest extension was $\sqrt{20}$. We again use a Gabriel graph to model the landscape connectivity, but note that alternative networks based on topographical data could be readily implemented.

To characterise environmental variability in dataset D, we followed the approach of

the authors of the original study and used the NIPALS (nonlinear estimation by iterative partial least squares) algorithm, which allows ordination of datasets including missing values [6], to extract the principal components of 14 environmental variables describing water chemistry, average climate, climate seasonality and variables describing the topography of landscape (see [7] for details). Dataset L did not include quantitative environmental variables. We therefore extracted elevation, mean annual temperature, and mean annual precipitation data from publicly available remote sensing databases (further details given in S4 Text).

Because the ecological interactions between the observed species are insufficiently known, we randomly sampled the interaction coefficients $\mathbf{A}_{ij}$ from the same discrete distribution used in the random metacommunities. For a community model with no spatial extent for which $P(A_{ij} = 0.3) = 0.3$, and otherwise $A_{ij} = 0$, the onset of ecological structural instability occurs when $S_0 \approx 40$. From the SAR the effective area of a given site can be estimated using the equation

$$a_x = a_0 \left( \frac{S_x}{S_0} \right)^{\frac{1}{z}}, \tag{S6}$$

where we assume $z = 1$ as explained above. Substituting Eq. S6 into Eq. 2, the scaling of the competitive overlap matrix for the fitted metacommunity model can be written as

$$\mathbf{A}_x = \left( \frac{S_x}{S_0} \right)^{\frac{v}{z}} \mathbf{A}_0 + \left( \frac{S_x}{S_0} \right)^{\frac{v'}{z}} \mathbf{I}, \tag{S7}$$

where $S_x$, the observed richness at a given site, is extracted from the empirical data.

When assembling fitted metacommunity models, we again made the simplifying assumption that abiotic niche widths $w$ are identical for both environmental variables and all species. Greater accuracy in model fitting could be achieved by estimating the typical abiotic niche widths of each variable independently, and by incorporting inter-specific variation in niche width.

For simulated LVMCM metacommunities with spatial, environmental and local richness distributions matching empirical observations we fit the parameter $w$ based on the match between the observed and simulated occupancy frequency distribution (OFD) [8], the proportion of species occupying a given number of sites (ranging from 1 for the site with the highest estimated conservation value to $N$ the number of sites in the dataset). Since we find the parameter $\ell$ to have very little impact on the response to site removal, this was kept fixed at an intermediate value when fitting to observations. We use an unconventional approach to quantitative model fitting in which we fit the patch occupancy model outlined in S3 Text to both empirical observation and simulated metacommunities. This model has a single fitting parameter $m$ and therefore can be readily fit to data. We first estimated this parameter for the empirical datasets, denoted $m_{\texttt{EMP}}$. Then, for LVMCM models with key properties of the empirical systems used to define the abiotic environment, we varied the niche width parameter $w$ and for each assembly estimated fitting parameter of the patch occupancy model, $m_{\texttt{SIM}}$. Finally, we selected as the best fitting simulation, those with niche width $w$ for which the absolute difference between $m_{\texttt{EMP}}$ and $m_{\texttt{SIM}}$ was minimised. Note that previous work has demonstrated the ability of the LVMCM to reproduce a variety of key macroecological patterns [1,9]. In parallel work, we are rigorously testing the biological plausibility of the patch occupancy model. Thus, while an unconventional approach, both models have been systematically tested and found to be well supported.

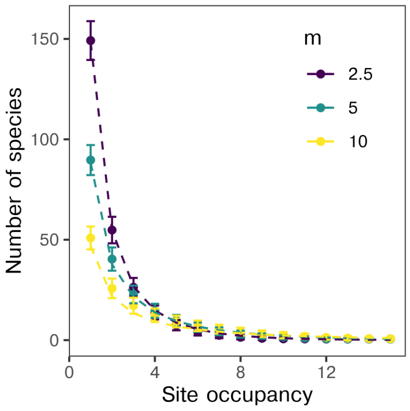


**Fig C. The steady state OFD of the locally saturated patch occupancy model.** Points represent the average number of species in each site occupancy for 1000 realisations of the LSPOM described in the text for three different values of the mixing rate $m$. Dashed lines are the predicted OFDs computed by numerically solving Eq. (S8).

## C Remote sensing data collection

We extracted temperature, precipitation and elevation data for the 23 sites of the Brazilian Lichen-Fungi survey [10]. The first two abiotic variable were time averaged over the 20 years from January 2000 to January 2020. We pooled nearby sites in order to reduce the dimensionality and therefore the computational load of the simulations. For these sites we took abiotic values, including spatial coordinates, as the between site averages.

Temperature data were collected from the MODIS satellite database [11]. For each site, sensor products were filtered by land surface temperature and emissivity to retrieve data from the MODIS thermal infrared bands. Precipitation data were collected from the IMERG satellite database [12]. Elevation data were taken from the GTOPO30 digital elevation model database [13] which provides data from collective satellite inputs, compiled by the U. S. Geological Survey.

## D Patch occupancy fitting model

We use a simple locally saturated patch occupancy model (LSPOM) as an intermediary for comparison of the occupancy frequency distributions (OFD) of LVMCM and empirical datasets. The LSPOM conceptualizes a metacommunity as a spatially implicit species-by-site occupancy matrix describing an assemblage of $S$ species occupying $N$ patches. The $i$th patch can host up to $\alpha_i$ populations without losing species due to ecological structural instability, such that in total the metacommunity can host up to $Z = \sum_i^N \alpha_i$ local populations. The model assumes that this upper limit is attained, i.e. that communities are 'locally staturated'.

At each time step of the model a new species invades the metacommunity from outside and colonizes a single site. Simultaneously, each extant local population colonises with probability $m/Z$ an additional site, where $m$ is the mixing rate. In both processes, the site $i$ colonized by a species is chosen at random with probability $\alpha_i/Z$. Each colonisation event, either from within or from outside the metacommunity, produces a corresponding extirpation of a randomly selected occupant of the colonised site, in accordance with the assumption of local ecological saturation.

For $N \gg m$, the steady state OFD for this process is such that the mean number of

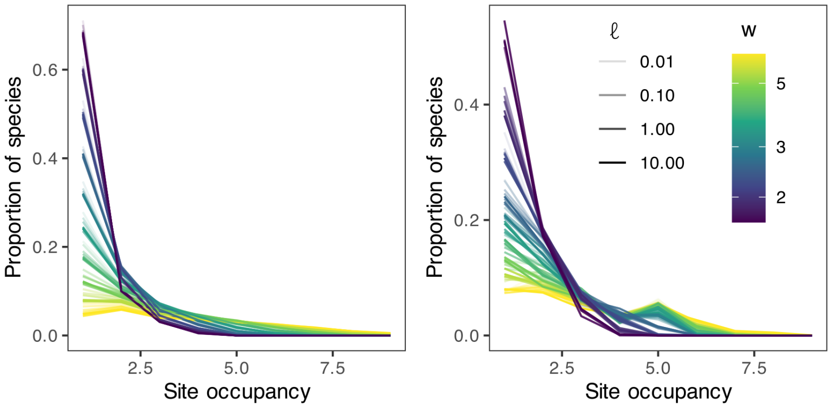


**Fig D. The impact of parameter variation on the OFD for fitted models.** The average proportional occupancy frequency distributions for various values of $w$ with landscape properties extracted from empirical datasets L and D. Colour represents abiotic niche width $w$.

species occupying $n$ patches is approximately

$$\frac{Z}{m\,n}\left(\frac{m}{m+1}\right)^n . \tag{S8}$$

In Fig C we show the log-series steady state of a numerical formulation of the LSPOM.

To find the best fitting abiotic niche width $w$ for an empirical dataset, we estimated the mixing rate $m$ for the empirical OFD, then, for model metacommunities with abiotic variables extracted from observation data as described above, we scanned assembled metacommunities with various combinations of the focal parameters (Fig D). The best fitting $w$ was selected as that with the emergent OFD that closest matched the mixing rate $m$ of the observation. Note that, for simplicity we define the site occupancy in the LVMCM with reference to source populations only. These are the local populations which persist following a long relaxation ($10^4$ unit times) after dispersal is switched off. In effect this assumes that only source populations are detected in the empirical data due to type II sampling errors. Alternative approaches to defining local site occupancy in simulations are available but typically require additional model assumptions or setting an arbitrary numerical threshold.

# Bibliography


1. O'Sullivan JD, Knell RJ, Rossberg AG. Metacommunity-Scale Biodiversity Regulation and the Self-Organised Emergence of Macroecological Patterns. Ecology Letters. 2019;22(9):1428–1438. doi:10.1111/ele.13294.

2. Gilbert B, Tunney TD, McCann KS, DeLong JP, Vasseur DA, Savage V, et al. A Bioenergetic Framework for the Temperature Dependence of Trophic Interactions. Ecology Letters. 2014;17(8):902–914.

3. Allen AP, White EP. Effects of range size on species–area relationships. Evolutionary Ecology Research. 2003;5(4):493–499.

4. Rosindell J, Cornell SJ. Species–area relationships from a spatially explicit neutral model in an infinite landscape. Ecology letters. 2007;10(7):586–595.

5. Storch D. The theory of the nested species–area relationship: geometric foundations of biodiversity scaling. Journal of Vegetation Science. 2016;27(5):880–891.

6. Ibáñez C, Alcaraz C, Caiola N, Rovira A, Trobajo R, Alonso M, et al. Regime shift from phytoplankton to macrophyte dominance in a large river: top-down versus bottom-up effects. Science of the Total Environment. 2012;416:314–322.

7. Benito X, Fritz SC, Steinitz-Kannan M, Vélez MI, McGlue MM. Lake regionalization and diatom metacommunity structuring in tropical South America. Ecology and evolution. 2018;8(16):7865–7878.

8. McGeoch M, Gaston KJ. Occupancy frequency distributions: patterns, artefacts and mechanisms. Biological Reviews. 2002;77(3):311–331.

9. O'Sullivan JD, Terry JCD, Rossberg AG. Intrinsic ecological dynamics drive biodiversity turnover in model metacommunities. Nature Communications. 2021;doi:10.1101/2020.05.22.110262.

10. da Silva Cáceres ME, Aptroot A, Lücking R. Lichen fungi in the Atlantic rain forest of Northeast Brazil: the relationship of species richness with habitat diversity and conservation status. Brazilian Journal of Botany. 2017;40(1):145–156.

11. Hulley G, Hook S. MOD21A2 MODIS/Terra Land Surface Temperature/3-Band Emissivity 8-Day L3 Global 1km SIN Grid V006. NASA EOSDIS Land Processes DAAC. 2017;doi:https://doi.org/10.5067/MODIS/MOD21A2.006.

12. Huffman G, Bolvin D, Braithwaite D, Hsu K, Joyce R, Xie P. Integrated Multi-satellitE Retrievals for GPM (IMERG), version 4.4. NASA's Precipitation Processing Center. 2014;.

13. Danielson JJ, Gesch DB. Global multi-resolution terrain elevation data 2010 (GMTED2010). US Department of the Interior, US Geological Survey; 2011.

## Supporting Information Legends

S1 Text
