# Supplementary material for: Community composition exceeds area as a predictor of long-term conservation value
Source: PLoS Comput Biol. 2023 Jan 30;19(1):e1010804. doi: 10.1371/journal.pcbi.1010804 (PMC9946215; doi:10.1371/journal.pcbi.1010804)
Supplement: Attachment — Submitted filename: Editorial checklist.odt [file pcbi.1010804.s002.odt]

Manuscript ID: PCOMPBIOL-D-22-00580R1 


Dear Dr O'Sullivan, 

Thank you for submitting your paper, "Community composition exceeds area as a predictor of long-term conservation value", to PLOS Computational Biology. We have checked over your submission and, before we can assign your manuscript to the Editor, we ask that you first please make the following modifications: 

1) We ask that a manuscript source file is provided at Revision. Please upload your manuscript file as a .doc, .docx, .rtf or .tex. If you are providing a .tex file, please upload it under the item type ‘LaTeX Source File’ and leave your .pdf version as the item type ‘Manuscript’.

Done

2) Please ensure that the CRediT author contributions listed for every co-author are completed accurately and in full. At this stage, "All Authors" require contributions. Please ensure that the full contributions of each author are acknowledged in the ""Add/Edit/Remove Authors"" section of our submission form. 

The list of CRediT author contributions may be found here: 
https://journals.plos.org/ploscompbiol/s/authorship#loc-author-contributions

Done

3) Please ensure that all author names are spelled correctly in the manuscript file, including the use of a middle name/initials, and any special characters (accents, umlauts, etc.). Please place first/given names before last/family names. All authors must have a first and last name. We noticed that "Jacob O'Sullivan" is spelled differently between the manuscript file and submission form. Please be aware that correct spelling and formatting of author names in the article file is vital for accurate indexing in PubMed. 

Done

4) Please provide an Author Summary. This should appear in your manuscript between the Abstract (if applicable) and the Introduction, and should be 150–200 words long. The aim should be to make your findings accessible to a wide audience that includes both scientists and non-scientists. Sample summaries can be found on our website under Submission Guidelines: 
https://journals.plos.org/ploscompbiol/s/submission-guidelines#loc-parts-of-a-submission 

Done

5) Please upload all main figures (Fig1 to Fig 6) as separate Figure files in .tif or .eps format. For more information about how to convert and format your figure files please see our guidelines: 
https://journals.plos.org/ploscompbiol/s/figures

Done

6) We have noticed that you have uploaded Supporting Information file, but you have not included a list of legends. Please add a full list of legends for your Supporting Information files after the references list. 

Done

7) Please amend your detailed Financial Disclosure statement. This is published with the article, therefore should be completed in full sentences and contain the exact wording you wish to be published. 
i). State the initials, alongside each funding source, of each author to receive each grant. 
ii). State what role the funders took in the study. If the funders had no role in your study, please state: “The funders had no role in study design, data collection and analysis, decision to publish, or preparation of the manuscript.” 
iii). If any authors received a salary from any of your funders, please state which authors and which funders. 

Done

8) In the online submission form, you indicated that your data will be submitted to a repository upon acceptance. We strongly recommend all authors deposit their data before acceptance, as the process can be lengthy and hold up publication timelines. Please note that, though access restrictions are acceptable now, your entire data will need to be made freely accessible if your manuscript is accepted for publication. This policy applies to all data except where public deposition would breach compliance with the protocol approved by your research ethics board. If you are unable to adhere to our open data policy, please kindly revise your statement to explain your reasoning and we will seek the editor's input on an exemption. Please be assured that, once you have provided your new statement, the assessment of your exemption will not hold up the peer review process. 

As previously stated, simulation data will be uploaded upon acceptance. As also noted in the manuscript, the source code for the simulation will also be make public at this stage.

9) Some material included in your submission may be copyrighted. According to PLOS’s copyright policy, authors who use figures or other material (e.g., graphics, clipart, maps) from another author or copyright holder must demonstrate or obtain permission to publish this material under the Creative Commons Attribution 4.0 International (CC BY 4.0) License used by PLOS journals. Please closely review the details of PLOS’s copyright requirements here: PLOS Licenses and Copyright. If you need to request permissions from a copyright holder, you may use PLOS's Copyright Content Permission form. 

Replaced figure with open source map (Stamen).

Please respond directly to this email and provide any known details concerning your material's license terms and permissions required for reuse, even if you have not yet obtained copyright permissions or are unsure of your material's copyright compatibility. Once you have responded and addressed all other outstanding technical requirements, you may resubmit your manuscript within Editorial Manager. 

Potential Copyright Issues: 

Figure [2]: please (a) provide a direct link to the base layer of the map (i.e., the country or region border shape) and ensure this is also included in the figure legend; and (b) provide a link to the terms of use / license information for the base layer image or shapefile. We cannot publish proprietary or copyrighted maps (e.g. Google Maps, Mapquest) and the terms of use for your map base layer must be compatible with our CC BY 4.0 license. 

Note: if you created the map in a software program like R or ArcGIS, please locate and indicate the source of the basemap shapefile onto which data has been plotted. 

If your map was obtained from a copyrighted source please amend the figure so that the base map used is from an openly available source. Alternatively, please provide explicit written permission from the copyright holder granting you the right to publish the material under our CC BY 4.0 license. 

If you are unsure whether you can use a map or not, please do reach out and we will be able to help you. The following websites are good examples of where you can source open access or public domain maps: 
* U.S. Geological Survey (USGS) - All maps are in the public domain. (http://www.usgs.gov) 
* PlaniGlobe - All maps are published under a Creative Commons license so please cite “PlaniGlobe, http://www.planiglobe.com, CC BY 2.0” in the image credit after the caption. (http://www.planiglobe.com/?lang=enl) 
* Natural Earth - All maps are public domain. (http://www.naturalearthdata.com/about/terms-of-use/) 

Once you have made the necessary changes in Editorial Manager, please re-approve the submission by following the same steps as before. 

Please resolve these issues and resubmit your manuscript within 30 days. If you need more time, please email the journal office at ploscompbiol@plos.org. We are happy to grant extensions of up to one month past this due date. If we do not hear from you within 30 days, we will withdraw your manuscript on your behalf.                                         

If you have any questions or require assistance, please don't hesitate to get in touch. To help us minimize inbox volumes, we kindly ask that you not reply to this email simply to confirm you have resubmitted your revisions. 

Kind regards, 

Nithya Venkatesan 
PLOS Computational Biology 
ploscompbiol@plos.org 

Author guidelines: https://journals.plos.org/ploscompbiol/s/submission-guidelines 

__________________________________________________ 
In compliance with data protection regulations, you may request that we remove your personal registration details at any time. (Use the following URL: https://www.editorialmanager.com/pcompbiol/login.asp?a=r). Please contact the publication office if you have any questions.
